# Association of 5-lipoxygenase gene polymorphisms with bronchial asthma

**DOI:** 10.3892/etm.2012.712

**Published:** 2012-09-17

**Authors:** CHUNYING BAI, XIAOMING YU, RUI YUN, TIEWEI SHI, CHAOJUN ZHANG, JING ZHOU, SACHURANGUI, LIJUAN TONG, XIUJUN LI, LIFENG GAO

**Affiliations:** 1 Molecular Medicine Research Center, School of Medical Science, Chifeng University;; 2 Departments of Pediatrics and; 3 Pneumology, First Affiliated Hospital of Chifeng University;; 4 Department of Pediatrics, Chifeng City Hospital, Chifeng, Inner Mongolia, P.R. China

**Keywords:** single-nucleotide polymorphism, 5-lipoxygenase, bronchial asthma

## Abstract

Leukotrienes are important pro-inflammatory mediators in bronchial asthma (BA) and are derived from arachidonic acid by the action of 5-lipoxygenase (5-LO). We investigated the association of 5-LO gene polymorphisms with BA. Thirty-six single-nucleotide polymorphisms (SNPs) of the 5-LO gene, as referenced in the dbSNP gene bank, were analyzed with sequencing and allele-specific PCR (AS-PCR) in genomic DNA from individuals with BA and controls. Of these 36 SNPs, 4 were identified in our study. The c.760 G>A (E254K) (rs2228065) was detected in 15 out of 215 BA patients and 6 out of 212 controls (P<0.05). There were no differences in the frequencies of the other three silent polymorphisms, rs2228064 (c.270 G>A), rs116961353 (c.780G>A) and rs2229136 (c.1728 A>G) between individuals with BA and controls (P>0.05). With our designed primers for AS-PCR, the detection of the 5-LO gene E254K polymorphism was clear and accurate, and the genotype was directly distinguished. Our findings contribute to the evaluation of one of the genetic risk factors for BA and we report an accurate and simple method to rapidly detect the 5-LO E254K polymorphism. It is important to further study the correlation between drug response in BA patients using 5-LO inhibitors with the E254K polymorphism in the clinic.

## Introduction

The incidence of asthma in numerous countries is increasing and the cause of the disease is complex (1,2). Leukotrienes (LTs) play an important pro-inflammatory role in both early-and late-phase asthmatic responses (3). LTs constitute a class of potent biological mediators of inflammation and anaphylaxis. 5-Lipoxygenase (5-LO) is an essential enzyme which catalyzes the first committed steps in the biosynthetic pathway leading to the production of LTs (4–6). The 5-LO gene is located on chromosome 10q11.2 (7). In several studies, the addition of an Sp-1 binding motif (-GGGCGG-) or the deletion of one or two Sp-1 binding motifs in the 5-LO core promoter have been shown to be associated with reduced gene expression (8,9). Our previous study revealed that the E254K polymorphism in the 5-LO gene may be associated with bronchial asthma (BA) in Japanese children (10). However, few studies have investigated the role of polymorphisms of the 5-LO gene in a Chinese population. Therefore, it is necessary to identify such single-nucleotide polymorphisms (SNPs) in the 5-LO gene and to research the correlation between SNPs in the 5-LO gene and the incidence of BA in the Chinese population.

## Patients and methods

### Patients and control subjects

A total of 179 Chinese Han children with BA (112 males and 67 females; mean age, 2.73±10.27 years), 36 adults with BA (20 males and 16 females; mean age, 51±28 years), 50 Chinese Mongolian children with BA (32 males and 18 females; mean age, 2.86±10.14 years), 50 non-allergic control Han children (30 males and 20 females; mean age, 2.82±10.18 years) and 162 Han adult controls (90 males and 72 females; mean age, 19.7±2.3 years) were enrolled. The diagnosis of BA was made according to the criteria of the American Thoracic Society (11). The controls were healthy and did not have a history of allergic diseases. All subjects were randomly selected among patients in our hospital or our college. Informed consent was obtained from all individuals or their parents.

### Peripheral blood collection and separation of cells

Peripheral blood (2 ml) was obtained from each patient and treated with 0.2% NaCl to collect leukocytes. Genomic DNA was extracted from the leukocytes with a DNA Extract kit (Bioteke, Beijing, China) (12).

### Allele-specifc (AS)-PCR primer design

AS-PCR is selective PCR amplification of one allele and is used to detect SNPs. Selective amplification is usually achieved by designing a primer such that it will match/mismatch one of the alleles at the 3′ end and the penultimate base is designed to incorporate the SNP in order to improve the specificity of the primer. We designed the following primers: P1, 5′-cgc tgc aca gag ctg cct g (254Glu); P2, 5′-cgc tgc aca gag ctg cct a (254Lys); P3, 5′-cgc aat tcc tcc tct gat gt (co-reverse primer); PCR product, 301-bp. We also designed a pair of reference primers: P4, 5′-aga ggc gaa gtt ctc caa ca; P5, 5′-aac agg gac gga gag tga tg; PCR product, 600-bp. The primer for c.780 G>A (c.780G) was 5′-aga agc tcc cgg tga cca tg-3′ and that for c.780 G>A (c.780A) was 5′-aga agc tcc cgg tga cca ta-3′.

### AS-PCR system and PCR conditions

P3, P4 and P5 primers were added to both A and B tubes (P1,P3,P4 and P5 primers in A tube; P2,P3,P4 and P5 primers in B tube). A and B tubes were prepared and added to 2 μl (10 μM) wild-type P1 and mutant P2 primers, respectively. Two tubes were added per 1 μl (10 μM) a pair of reference primers, P4 and P5, as well as 2 μl (10 μM) common reverse primer P3 were also used. The reaction mixture consisted of 25 μl 2X PCR Mastermix (Bioteke, Beijing, China), 1 μl genomic DNA (100-150 ng/μl) and 18 μl sterilized water in a total volume of 50 μl. The PCR conditions were as follows: 95°C for 5 min, then 95°C for 30 sec, 56°C for 30 sec and 72°C for 1 min for 35 cycles, the last cycle after 72°C was extended for 10 min. The products were stored at 4°C.

### Detection of SNPs in the 5-LO gene by sequencing

The 14 exons of the 5-LO gene were amplified using the PCR technique and sequenced using an ABI 3100 DNA auto-sequencer (Applied Biosystems, Carlsbad, CA, USA) in individuals with BA (n=30) and controls (n=30). For further study, the E254K and c.780 G>A substitution was detected by AS-PCR in all individuals with BA (n=215) and controls (n=212) and the other two silent polymorphisms (c.270 G>A, c.1728 A>G) were detected in 65 individuals with BA and 56 controls. The primer details for the PCR used in the detection of these polymorphisms are shown in Table I.

### Statistical analyses

Allele and genotype frequencies were calculated for each locus and tested for Hardy-Weinberg equilibrium. The distribution of the genotype of E254K in the 5-LO gene was analyzed by Fisher’s exact test. P<0.05 was considered to indicate a statistically significant result. The significance of the differences in clinical characteristics was analyzed using the two-sample t-test.

## Results

### Polymorphisms in the 5-LO gene

**Figure 1:**
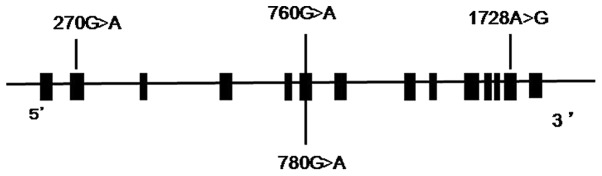
Gene structure and polymorphisms investigated in the 5-LO gene. The positions marked in bold were found in polymorphisms in the Chinese Han population. 5-LO, 5-lipoxygenase.

We identified 4 SNPs in the 5-LO gene in individuals with BA (Fig. 1). Three SNPs were silent polymorphisms [c.270 G>A (exon 2), c.780 G>A (exon 6) and c.1728 A>G (exon 13)]. There were no differences in the frequencies of these three SNPs between individuals with BA and controls (Table II).

One SNP was a missense polymorphism and the amino acid at 254 was changed from Glu (E) to Lys (K) (rs2228065). We determined the prevalence of c.760 G>A (E254K) in the 5-LO genes of individuals with BA and controls. This SNP was found in 15 (8 males and 7 females; mean age, 2.19±4.81 years; 0.070) out of the 215 individuals with BA and 6 (0.028) of the 212 controls. The mutant allele frequency was 0.035 in 215 individuals with BA and 0.014 in the 212 controls (Table III). There was a significant difference in the E254K frequency between individuals with BA and controls (Fisher’s exact test, P<0.05). We detected the E254K polymorphism in 50 Chinese Mongolian BA patients by AS-PCR and it was present in 3 patients.

### Agarose gel electrophoresis

**Figure 2:**
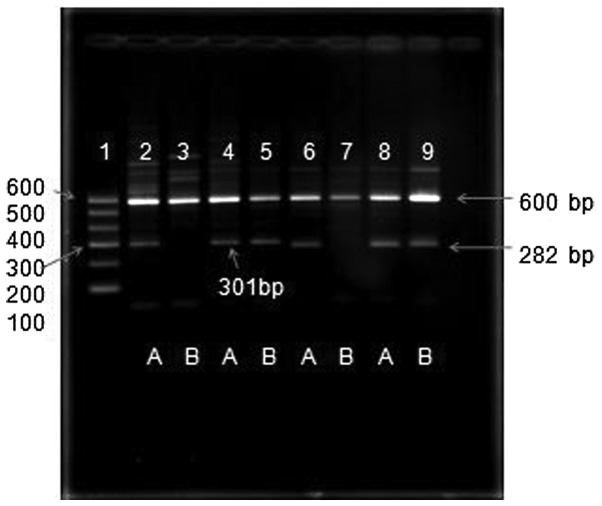
Agarose gel electrophoresis results of 5-LO E254K and c.780G>A polymorphisms by AS-PCR. 1, marker I; 2 and 3, 254 wild-type GG; 4 and 5, 254 heterozygous type GA; 6 and 7, c.780 wild-type GG; 8 and 9, c.780 heterozygous type GA. 5-LO, 5-lipoxygenase; AS-PCR, allele-specific PCR.

DNA marker I (600, 500, 400, 300, 200 and 100 bp; 5 μl) and 10 μl AS-PCR products were analyzed by 2% agarose gel electrophoresis. With our designed primers for AS-PCR, it was possible to accurately and clearly detect the 5-LO gene rs2228065 (E254K) and rs116961353 (c.780G>A) polymorphisms, and the genotype was directly distinguished (Fig. 2). Compared with the sequencing and restriction enzyme method, this method is economical, quick and simple.

### Associations of E254K with clinical characteristics

We compared the clinical characteristics with the results of a routine blood test by automatic blood analyser (Sysmex 1800i;. Sysmex Corp., Kobe, Japan) among the controls and patients with BA with and without E254K (Table IV). The percentages of lymphocytes and monocytes and the levels of platelets were significantly higher in individuals with BA (without or with E254K) than in controls (P<0.05). However, the percentage and number of neutrophils were significantly lower in individuals with BA (without or with E254K) than in the controls (P<0.05). No significant difference was identified between the clinical features in BA patients with E254K compared with those without E254K. The percentage of eosinophils (EO%) was significantly lower in individuals with BA and E254K than in controls (P=0.04). There was no significant difference in age or gender between BA patients with and without E254K (P>0.05).

## Discussion

Allergic diseases, including asthma and atopic dermatitis, are complex genetic disorders that do not conform to a simple Mendelian pattern of inheritance (13). The etiology is extremely complex and the incidence of asthma is correlated with environmental and genetic factors. Clinically similar asthma symptoms may be caused by different mechanisms (14). In this study, we studied polymorphisms of the 5-LO gene and attempted to investigate the association of 5-LO gene polymorphisms with BA in a Chinese Han population. We compared the difference in the 5-LO gene polymorphism frequency between asthma patients and normal controls and evaluated the correlation of the SNPs with asthma onset, age, gender, severity of asthma and other clinical characteristics of BA. We found a missense SNP and three silent SNPs in the 5-LO gene in BA patients.

Our study showed that the c.760 G>A (E254K) polymorphism in the 5-LO gene was associated with BA in a Chinese Han population. There was a significant difference in the E254K frequency between individuals with BA and controls (Fisher’s exact test, P<0.05). Three other silent SNPs, rs2228064 (c.270 G>A), rs116961353 (c.780G>A) and rs2229136 (c.1728 A>G), described previously (15), were also identified, but there were no significant differences in the frequencies between individuals with BA and controls (P>0.05). We also detected the E254K polymorphism in Chinese Mongolian BA patients by AS-PCR; there were 3 heterozygotes out of 50 patients. Our results indicate that the 5-LO E254K polymorphism is present in patients with BA in Japanese (in our previous research), Chinese Han and Chinese Mongolian populations. The E254K allele and genotype frequencies were significantly different in individuals with BA compared with the control group. Our study further confirmed that the 5-LO E254K polymorphism may be correlated with BA.

To examine the functional effects of c.760 G>A (E254K) in the 5-LO gene, we compared the clinical characteristics with results from routine blood test reports performed in the last three years in our hospital among the controls, BA with E254K and BA without E254K. However, the clinical features (including the age and gender) in our study were not significantly different between individuals with BA with and without E254K (P>0.05). This result may be due to the number of individuals with BA and E254K being too low to obtain accurate statistical results.

The percentages of lymphocytes, monocytes and platelets (10^9^/l) levels were significantly higher in individuals with BA (without or with E254K) than in controls (P<0.05). Monocytes are the largest cells in normal blood. They act as phagocytes in certain inflammatory diseases and are the body’s second line of defense against infection (16). Lymphocytes are the primary components of the body’s immune system and increase in number in numerous viral infections and with tuberculosis (17). Inflammatory disorders may cause a high platelet count in a similar way to infections. Our date support the theory that inflammation is a cause of asthma attack.

The percentage and number of neutrophils were significantly lower in individuals with BA (without or with E254K) than in controls (P<0.05). A decrease in neutrophil levels is known as neutropenia. Although most bacterial infections stimulate an increase in neutrophils, certain bacterial infections, including typhoid fever and brucellosis, and numerous viral diseases, including hepatitis, influenza, rubella, rubeola and mumps, decrease the neutrophil count. Our data support the hypothesis that viral infection is a cause of asthma attack.

The mean level of eosinophils showed a tendency to increase in individuals with BA without E254K more than in controls. However, the percent eosinophil levels were significantly lower in individuals with BA and E254K than in controls (P=0.04). Eosinophils are associated with antigen-antibody reactions (18). The most common reasons for an increase in the eosinophil count are allergic reactions such as hay fever, asthma or drug hypersensitivity. Decreases in the eosinophil count may be observed when a patient is receiving corticosteroid drugs.

Pharmacogenetics is the study of how genetic differences influence the variability of patients’ responses to therapy (19). Therefore, we intend to study the correlation between these four SNPs and patients’ responses to therapy. With our designed primers for AS-PCR, the detection of the 5-LO gene E254K and c.780G>A polymorphisms is accurate and clear and the genotype may be directly distinguished. Compared with the sequencing and restriction enzyme method, our method was economic, quick and simple.

In summary, our findings contribute to the evaluation of one of the genetic risk factors for BA and we report an accurate and simple method to quickly detect the 5-LO E254K polymorphism. It is important to further study the correlation between the response to 5-LO inhibitors with the E254K polymorphism in the clinic. These results are important for clarifying the different mechanisms of BA.

## Figures and Tables

**Table I:** Primer details for PCR to detect 4 polymorphisms by sequencing.

Primer	Sequence	Amplified product size (bp)	Annealing temperature (°C)
c.270 G>A FP	5′ CTC CAG AAC AAA GGC TCA GG 3′	360	56
c.270 G>A RP	5′ CCT GCA CAG CAG TGT CAT TC 3′		
c.760 G>A (E254K) FP	5′ GCA GGG ACT CTG CTC TTA GG 3′	473	56
c.760 G>A (E254K) RP	5′ CGC AAT TCC TCC TCT GAT GT 3′		
c.780 G>A FP	5′ GCA GGG ACT CTG CTC TTA GG 3′	473	56
c.780 G>A RP	5′ CGC AAT TCC TCC TCT GAT GT 3′		
c.1728 A>G FP	5′ GAA AGA GGA TGG ACG GAC TG 3′	295	55
c.1728 A>G RP	5′ CTC GTT TTC CTG GAA CTG GC 3′		

FP, forward primer; RP, reverse primer.

**Table II:** Allele and genotype frequencies of 5-LO silent SNPs in a Chinese Han population.

	Frequency of 5-LO SNPs		
Allele/genotype	Non-allergic controls	Bronchial asthma patients	P-value
Allele			
c.270 G>A	n=56	n=65	
G	101 (0.902)	118 (0.908)	>0.05
A	11 (0.098)	12 (0.092)	
c.780 G>A	n=212	n=215	
G	413 (0.974)	417 (0.970)	
A	11 (0.026)	13 (0.030)	>0.05
c.1728 A>G	n=56	n=65	
A	105 (0.938)	126 (0.969)	>0.05
G	7 (0.058)	4 (0.033)	
Genotype			
c.270 G>A	n=56	n=65	
GG	45 (0.804)	53 (0.815)	>0.05
GA	11 (0.196)	12 (0.185)	
AA	0	0	
c.780 G>A	n=212	n=215	
GG	201 (0.948)	202 (0.940)	
GA	11 (0.052)	12 (0.056)	
AA	0 (0)	1 (0.005)	>0.05
c.1728 A>G	n=56	n=65	
AA	46 (0.821)	54 (0.831)	>0.05
AG	10 (0.179)	11 (0.169)	
GG	0	0	

5-LO, 5-lipoxygenase; SNP, single-nucleotide polymorphism.

**Table III:** Allele and genotype frequencies of 5-LO missense SNP in a Chinese Han population.

	Frequency of 5-LO SNP		
Allele/genotype	Non-allergic controls (n=212)	Bronchial asthma patients (n=215)	P-value
c.760 G>A			
Allele			
G	418 (0.986)	415 (0.965)	
A	6 (0.014)	15 (0.035)	<0.05
Genotype			
GG	206 (0.972)	200 (0.930)	
GA	6 (0.028)	15 (0.070)	<0.05
AA	0	0 (0)	

5-LO, 5-lipoxygenase; SNP, single-nucleotide polymorphism.

**Table IV:** Clinical characteristics of the controls, BA patients with E254K and BA patients without E254K.

Clinical characteristics	Normal range	Controls (n=50) mean ± SD	BA with E254K (n=14) mean ± SD	BA without E254K (n=114) mean ± SD	P-value (t-test)		
					P1	P2	P3
WBC (10^9^/l)	4–10	8.07±1.6	7.86±3.49	8.79±3.7	0.852	0.445	0.496
LYMPH%	20–40	30.94±15.72	61.72±15.71	54.81±18.72	**2.89E-6**	**1.79E-6**	0.314
MONO%	3–8	5.62±1.6	8.88±1.81	9.2±3.54	**2.82E-4**	**1.29E-4**	0.807
NEUT%	50–75	60.34±8.56	28.15±16.25	33.97±19.72	**4.01E-6**	**6.50E-7**	0.42
EO%	1–3	1.78±0.55	1.07±0.98	2.09±3.16	**0.040**	0.698	0.369
BASO%	0–1	0.25±0.16	0.26±0.2	0.31±0.3	0.888	0.450	0.666
LYMPH# (10^9^/1)	0.8–4	3.38±1.26	4.95±2.89	4.85±3.03	0.091	0.059	0.931
MONO# (10^9^/1)	0.12–1.8	0.61±0.38	0.66±0.38	0.80±0.44	0.742	0.094	0.365
NEUT# (10^9^/1)	2–7.5	4.86±2.35	2.16±1.35	2.92±2.14	**0.009**	**0.001**	0.325
EO# (10^9^/1)	0–0.45	0.11±0.10	0.08±0.07	0.18±0.27	0.426	0.351	0.310
PLT (10^9^/l)	100–300	238.63±65.08	333.38±129.46	320.35±106.32	**0.032**	**0.004**	0.744

P1, P-value comparing individuals with BA with E254K and controls; P2, P-value comparing individuals with BA without E254K and controls; P3, P-value comparing individuals with BA with E254K and BA without E254K. WBC, white blood cell; LYMPH, lymphocytes; MONO, monocytes; NEUT, neutrophils; EO, eosinophils; PLT, platelets; BA, bronchial asthma. The percentages of lymphocytes, monocytes and platelets levels were significantly higher in individuals with BA (without or with E254K) than in controls (P<0.05). By contrast, the percentage and number of neutrophils were significantly lower than in controls (P<0.05). P<0.05 in bold.

## Abbreviations:

SNP,: single-nucleotide polymorphism;
5-LO,: 5-lipoxygenase;
BA,: bronchial asthma;
LTs,: leukotrienes;
AS-PCR,: allele-specific PCR
